# Implementation of Anxiety UK’s Ask Anxia Chatbot Service: Lessons Learned

**DOI:** 10.2196/53897

**Published:** 2024-06-17

**Authors:** Luke Collins, Niamh Nicholson, Nicky Lidbetter, Dave Smithson, Paul Baker

**Affiliations:** 1 Linguistics and English Language Lancaster University Lancaster United Kingdom; 2 Anxiety UK Manchester United Kingdom

**Keywords:** chatbots, anxiety disorders, corpus linguistics, conversational agents, web-based care

## Abstract

Chatbots are increasingly being applied in the context of health care, providing access to services when there are constraints on human resources. Simple, rule-based chatbots are suited to high-volume, repetitive tasks and can therefore be used effectively in providing users with important health information. In this Viewpoint paper, we report on the implementation of a chatbot service called Ask Anxia as part of a wider provision of information and support services offered by the UK national charity, Anxiety UK. We reflect on the changes made to the chatbot over the course of approximately 18 months as the Anxiety UK team monitored its performance and responded to recurrent themes in user queries by developing further information and services. We demonstrate how corpus linguistics can contribute to the evaluation of user queries and the optimization of responses. On the basis of these observations of how Anxiety UK has developed its own chatbot service, we offer recommendations for organizations looking to add automated conversational interfaces to their services.

## Introduction

In the context of developing technologies, many businesses and services are turning to automated systems to provide users with information and accessible customer service. Among such tools, we find natural language processing systems, such as chatbots, that act as conversational interfaces, typically in lieu of interactions with human professionals. In health care, chatbots have a meaningful role to play, alongside other provisions, in increasing access to services, particularly in instances where there are restrictions in accessing face-to-face services [1,2]. Medical chatbots are already being used to provide and elicit information, create patient records, and discuss the results of clinical tests [3]. Furthermore, as Amiri and Karahanna [4] argue, health chatbots were shown to be particularly valuable in periods of quarantine as a response to the COVID-19 pandemic, in that “[t]heir scalability, wide accessibility, fast information dissemination, and substitution for in-person contact provide the functionality required to address the capacity expansion, social distancing requirements, and quick accurate information transmission needs of the public health response.” Ultimately, chatbot tools and similar automated systems can make an important contribution to the provision of health information and support in the context of time and resource restraints.

There is a wide range of capabilities demonstrated in the deployment of conversational interfaces of varying complexity, from rule-based chatbots that produce prewritten responses based on recognizing programmed terms and phrases to embodied conversational agents manifesting as a computer-generated avatar and smart conversational interfaces such as Apple’s Siri or Amazon’s Alexa [1]. Nevertheless, simple conversational agents are increasingly used in executing tasks without the need for human involvement, including booking appointments, purchasing merchandise, ordering food, and sharing information [5].

Research has shown that users respond positively toward the perceived convenience of medical chatbots, showing appreciation for swift information retrieval as an alternative to delays in scheduling a consultation, queuing in a phone service, or waiting for an email response [3]. There is wide acceptance of automated systems providing general health advice [2]. Furthermore, there are indications that computer services can reduce perceived stigma, in that users are more willing to disclose details about their health concerns to an automated system on the basis that they are regarded to be more trustworthy and nonjudgmental, reducing the potential for embarrassment [6,7]. In the mental health domain, chatbots and other kinds of conversational agents have been shown to assist in the diagnosis and reduction of symptoms among individuals with major depressive disorder, promote adherence, provide cognitive behavioral therapy, and cultivate a stronger therapeutic alliance compared with users’ interactions with a clinician [7].

This study details the development and learning from the implementation of a chatbot service through the website of the mental health charity Anxiety UK, the largest national charity in the United Kingdom to offer support for anxiety disorders. The charity has created a chatbot service called Ask Anxia to complement its other support and information services. In this study, we summarize the patterns of queries submitted to the Ask Anxia service after approximately 18 months of its activation using procedures from corpus linguistics, which involves using software tools to compute frequency-based measures of naturally occurring language data [8]. In addition, we review the quality of Ask Anxia’s responses based on manual coding. We offer some reflections on the development of the Ask Anxia service as “lessons learned,” with the intention that these will be instructive to others seeking to incorporate a conversational agent into their provision of information and support.

## Anxiety UK and Ask Anxia

Anxiety UK was established in 1970 and provides a wide range of support services and information for those affected by anxiety, stress, and anxiety-based depression. Anxiety disorders are characterized by excessive worry and fear [9] and are included among the “Common mental disorders” that are recorded as becoming increasingly prevalent in the United Kingdom [10]. The charity supports individuals from all over the United Kingdom and, in some cases, the rest of the world and has recently led on the development of an informal global alliance of not-for-profit anxiety organizations. Anxiety UK has a strong service delivery arm offering support via their helpline, therapy, peer support groups, and anxiety management courses. Most of, if not all, its volunteers, staff, and trustees have some experience of anxiety disorders. Anxiety UK states through all its communications, including the chatbot service, that it does not provide crisis support and directs those in need of such support to urgent care services such as the National Health Service and the charity, Samaritans.

Anxiety UK introduced an automated chatbot service, Ask Anxia, with the principal aim of offering an out-of-office-hours service to users, helping them to navigate more quickly to information that was already available, for example, through Anxiety UK’s web pages. Furthermore, the Anxiety UK team found that a high number of user queries received by phone or email concerned administration issues, and so, providing such information through an automated chatbot was seen as a way to release staff members and helpline volunteers to attend to other responsibilities that demanded more critical and engaged attention, including providing real-time interactional support via the helpline. Anxia is now a registered trademark that includes but is not limited to computer and application software provided by Anxiety UK as part of their mental health services.

Ask Anxia is a simple, pattern-matching chatbot that has been programmed to recognize certain stimuli (specific terms or phrases) and generate a response, which has been composed by the Anxiety UK team. At the time of writing, Ask Anxia had a content bank of 315 unique responses that has been developed and refined since the service has been operational, and the Anxiety UK team continues to monitor these response options based on the range of queries that users submit. An overview of the categories of responses is provided in Table 1, indicating the types of terms that Ask Anxia has been programmed to recognize in user queries.

Ask Anxia was launched in the beginning of July 2021, and we have applied procedures from corpus analysis (discussed in the Developments section) to 56 weeks’ worth of anonymized, aggregated user queries submitted to the service (up until the end of July 2022). This amounted to 14,359 queries consisting of 139,286 words.

**Table 1:** Recurring themes in queries to Ask Anxia and examples of terms used to determine a response.

Theme	Examples of pattern-matched terms
Request for help	help; support; advice; guidance
Looking for information on a specific anxiety type	GAD; health anxiety; OCD; PTSD; emetophobia; phobia
Physical symptoms	headache; chest pain; breathing; appetite; nausea; feel sick; dizzy
Psychological symptoms	intrusive thoughts; negative thinking; overthinking; constant worry
Information on a service	therapy; group; course; class; counselling; CBT; EMDR; resources
How to access a service	membership; cost; referral; book; sign up; join
Wanting to connect	talk; human; chat; agent
How to support others	family; partner; son; daughter; child; colleague
Getting involved with Anxiety UK	volunteering; approved therapist; fundraising; donate; placements
Diagnosis	do I have anxiety; diagnosis; symptoms
Medication	antidepressants; tablets; medication
Coping techniques	can't cope; what can I do; panic; now; thoughts; relax; sleep
Location	in person; areas; UK; Europe; face-to-face; online
Crisis	suicidal; self-harm; die

We recognize that there are important ethical considerations pertaining to the data, given that queries submitted to the Ask Anxia service are highly personal and relate to individuals’ well-being. In the privacy notice that is posted on the Anxiety UK website, users are informed that interactions with Ask Anxia are reviewed as part of the procedures for improving the quality of the service and that these may be shared with third parties for the purposes of research. Participants are discouraged from including personal information in their queries, and any such information that appears in the original message has been redacted. To protect the personal experiences of those who have accessed the service, we have provided generic examples, where cited, to demonstrate the interactional dynamics between constructed user queries and Ask Anxia’s (authentic) responses. Reported figures for word frequencies are based on original user queries. As part of a more recent update to the service (July 2022), a message encouraging users not to disclose personal information (such as name, address, and place of work) was added to the Ask Anxia header to ensure that this is visible. Furthermore, such information does not inform Ask Anxia’s pattern-matching programming, and so, it will only hinder the identification of an appropriate response.

In addition to reporting commonly used terms and phrases, we refer to the quality coding carried out by the Anxiety UK team, which is explained in the next section. Our study, then, offers a critical evaluation of the contribution of the chatbot Ask Anxia to Anxiety UK’s wider provision of services and helps us to understand the general patterns of what visitors to the site collectively seek, in terms of information and support. In the next section, we summarize the insights that we have gained through developing Ask Anxia’s programming, as the service has evolved over time.

## Developments

### Overview

In this section, we summarize the developments that have been applied to the Ask Anxia service based on observations of user queries, including where potential misunderstandings in queries asked by users arose. We present these developments as the lessons we have learned through reviewing the various updates that have been applied to the service since its launch, which are likely to be informative to those looking to implement similar tools. The continued monitoring of the service has contributed to its optimization and generated insights into user expectations. The time stamps for user queries indicate that 57.2% (8213/14,359) of the queries were submitted outside of Anxiety UK’s office hours (9:30 AM-5:30 PM), demonstrating that the Ask Anxia service is used when other contact services, such as the helpline, are closed. Indeed, one of the earliest modifications to Ask Anxia, in August 2021, was to remove the cap on how many queries it responded to, given its popularity.

### Updates Based on Frequent Terms in User Queries

The pattern-matched terms presented in Table 1 were largely informed by the Anxiety UK team’s own long-standing experiences of working with people seeking support for their, or a loved one’s, experiences of anxiety. Of course, the queries submitted to Ask Anxia provide further indications of what users seek from the service. As such, alongside the Anxiety UK team’s expert judgment, procedures from corpus linguistics can be drawn on to help identify topics and terms that are commonly cited by users, which can potentially highlight important areas for extending the existing information provision.

Corpus linguistics refers to a set or procedures for making quantitative and qualitative observations of the patterns of natural language use and can straightforwardly tell us, by way of a wordlist, for example, what the most common terms in our data are and how often they occur. We used the corpus analysis tool *#LancsBox* [11] to examine the user queries. Researchers have found, however, that because of how the English language is structured, often the most common words largely remain the same across data sets (typically, *I*, *the*, *you*, *and*, *it*, etc). Indeed, the 5 most frequent terms occurring in the user queries of the Ask Anxia service, were *I*, *to*, *a*, *and*, and *hi*. As such, corpus linguists have developed the concept of keyness, enabling us to determine which words appear in our data more frequently, to a statistically significant degree, when compared with a corpus of larger or equal size [8]. A keyness analysis of the queries submitted to Ask Anxia through comparison with a 10 million–word corpus of general English spoken language, the British National Corpus 2014 [12], identified the keywords that are particularly characteristic of the language used by contributors in this context. The statistical measure used in this case was log likelihood, which established a confidence score indicating that the observed differences are not the result of chance. A threshold value of 15.13 was applied, which equates with a *P* value of <.001. The top 20 keywords are shown in Table 2 and ranked according to log likelihood value (not reported).

**Table 2:** Keywords in user queries to the Ask Anxia service (n=139,286).

Rank	Keyword	Frequency, n (%)
1	anxiety	2461 (1.77)
2	hi	2670 (1.92)
3	help	1588 (1.14)
4	hello	1332 (0.96)
5	yeah	13 (0.01)
6	am	1123 (0.81)
7	anxious	525 (0.4)
8	therapy	451 (0.3)
9	my	2358 (1.69)
10	panic	373 (0.3)
11	support	411 (0.3)
12	im	302 (0.2)
13	membership	310 (0.2)
14	struggling	334 (0.2)
15	i	7805 (5.6)
16	ok	259 (0.2)
17	how	1370 (0.98)
18	oh	47 (0.03)
19	feeling	394 (0.3)
20	can	1606 (1.15)

What is clear from the keywords is the topical focus on *anxiety* and the prevalence of appeals for *help* and *support* on the basis that users are *struggling*, *feeling* *anxious*, or experiencing *panic* (attacks), for instance. We can also see that queries are typically written in the first person (*I, my,* and *im*), take a question form (*how* and *can*), and have a relatively informal style (*hi*, *yeah*, *ok*, and *im*), that is, consistent with the instant messaging–like format through which users interact with Ask Anxia.

The prevalence of the terms *help* and queries about *therapy* and *membership* indicate that the Anxiety UK team had largely anticipated the themes most often captured in user queries, as indicated in Table 1. Nevertheless, the recurrence of particular terms, including at specific moments, has informed the continued refinement of Ask Anxia’s responses and the information that is made available through the website. For example, in the week beginning September 13, 2021, keyness analysis showed that there was an increase in references to *fear* and *needle*, which coincided with booster doses of the COVID-19 vaccine being made available (to certain groups) and vaccines being approved for 12- to 15-year-olds, in anticipation of a new school term. Subsequently, the terms *fear* and *needle* appeared much more frequently in user queries. In response, Anxiety UK produced specific information concerning COVID-19 and related vaccines.

The Anxiety UK team has continued to extend Ask Anxia’s response options since it was launched in July 2021. In addition, because of identifying themes arising from user enquires, the team has carried out the following activities:

- created factsheets specifically on perinatal anxiety, peri- and postmenopausal anxiety, and negative thoughts and catastrophizing
- created additional web content such as adding a do-it-yourself self-diagnosis section to the “About Anxiety” page, extending the list of associated symptoms, explaining additional types of anxiety disorder such as dermatillomania, and adding further detail to the process of becoming a volunteer
- written and posted blogs on the topics of older people and anxiety, high functioning anxiety, highly sensitive people and anxiety, work anxiety, anxiety and appetite, autism and anxiety, anger and irritability with anxiety, returning to work post lockdown, and placements for students
- added entries to the frequently asked questions section relating to costs and arrangements for therapy
- extended member benefits, including researching the provision of fidget toys and fidget jewelry

As the updates have been informed by recurring user queries, we can expect that they will be of value to users generally, and by linking the updated information to Ask Anxia’s responses, a greater number of queries can be addressed automatically, out of hours and without the need for human intervention. Being able to identify trends in information-seeking requests has enabled Anxiety UK to respond operationally and strategically to meet the needs of its beneficiaries.

### Quality Coding

Each week, the Anxiety UK team manually coded a sample of Ask Anxia’s responses to monitor quality, which we have labelled Good, Okay, Bad, or Puzzled. On average, the Anxiety UK team would code 155 queries per week (ranging between 0 and 408). Good responses provided the appropriate information based on the query and constituted the response option that the human coder would have selected. The following example shows how Ask Anxia responds to the mention of “social anxiety” and directs users to the appropriate information:

Details about social phobia/social anxiety can be found here

link provided

Responses coded as Okay were not necessarily the optimal response option but were still topically relevant. For instance, in the case of a user posting a query that indicated that they wanted to talk to someone about dealing with anxiety, a human reader is likely to recognize the importance of talking to *someone*, whereas a response from Ask Anxia, which would subsequently be coded as Okay, might respond to the mention of anxiety as follows:

We provide a wide range of services and information for those dealing with anxiety, stress, or anxiety-based depression. Check out our homepage as a start here: [link provided] to see our calendar of upcoming events and our latest news.

In this instance, the user is still directed to information that is likely to be useful to them, even if this was not the primary purpose of their query. Where this points to a potential recalibration of the service is that Ask Anxia was already programmed with a response that more directly attends to the question of speaking with a (human) member of the team.

Bad responses appeared when there was misalignment with what the coders, and we, can perceive as the user’s intended meaning, with Ask Anxia generating an irrelevant or inappropriate response when a more pertinent option was available. For example, following a query that mentioned “joining,” that is, membership with Anxiety UK, Ask Anxia generated the following response pertaining to joining a webinar:

You can book on to our next webinar here:

link provided

Again, reviewing the cause of the misalignment highlights ways in which Ask Anxia can be improved.

Finally, certain responses are generated when a more specific alternative, relating to the topic or passage of interaction, is not available, such as:

Sorry, I am not sure how to answer that, do feel free to use our website search bar which may find the answer for you or contact our team directly; we are open Mon-Fri 09:30-17:30 (excluding bank holiday) and our contact list can be found here:

link provided

Such responses were coded as Puzzled.

Of the 14,359 queries, 8669 (60.37%) were subject to quality coding, and the distribution of these according to the different quality labels is shown in Table 3.

**Figure 1:**
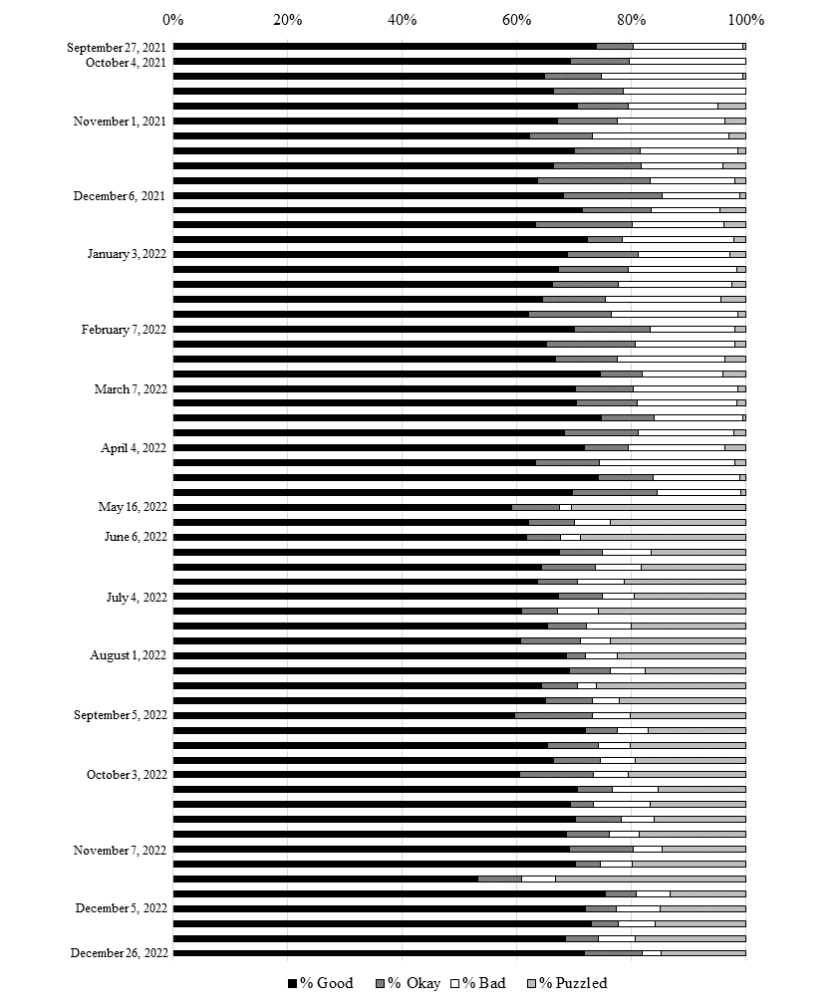
Quality coding of Ask Anxia responses as a percentage during each week from September 27, 2021, to December 26, 2022.

The quality coding figures provided in Table 3 indicate that Ask Anxia generally performed well, providing a Good response in two-thirds of cases. Furthermore, we can see how this coding was applied weekly, given that the Anxiety UK team made adjustments to Ask Anxia’s response options based on what they observed in their coding. Figure 1 indicates how the queries were coded between October 2021 and December 2022, showing the proportion of Ask Anxia responses that received the codes Good, Okay, Bad, and Puzzled.

**Table 3:** Number of queries coded according to each quality code (N=14,359).

Code	Queries, n (%)
Good	5801 (66.92)
Okay	911 (10.51)
Bad	1537 (17.73)
Puzzled	420 (4.84)

Figure 1 shows that the proportion of responses coded as Good was consistently >60% and that typically, Bad responses accounted for <30%. From May 2022 onward, there is a notable shift toward a smaller proportion of Bad and Okay responses and, instead, a greater number of Puzzled responses. As opposed to any change in the human evaluation of the responses or specific update to the software, this improvement reflects the ongoing work that the Anxiety UK team had been doing to calibrate the responses. This finding indicates that over time, Ask Anxia is less likely to generate an inappropriate or irrelevant response that could lead to disengagement from the user and more likely to provide a response that facilitates further engagement and opportunities for the user to find the relevant support.

The example of a Puzzled response provided earlier in this section on Quality coding was not initially one of the preprogrammed options but was added shortly after the launch (in September 2021), as the Anxiety UK team recognized a need to indicate when an optimal response could not be offered. The necessity of a Puzzled response is to be expected, given that the Anxiety UK team is not reasonably going to be able to anticipate the full range of queries users could conceivably submit. Furthermore, in many cases, a Puzzled response is preferable to a Bad response because it encourages the participant to remain engaged and try again. In work analyzing approximately 20,000 conversational exchanges between customers and a task-oriented chatbot for a Taiwanese banking firm, Li et al [13] focused on the problem of “nonprogress” responses, where users abandoned the dialogue. They identified a number of “reformulation” strategies when progress was halted, including rephrasing; adding different words; repeating the same words; and to a lesser extent, removing words [13]. This suggests that prompting the user to reformulate their query or to try an alternative mode of engagement, which the Puzzled response does, is preferable to closing down the exchange. Often, users simplify their reformulated messages [14], which increases the probability for pattern matching and Ask Anxia finding a relevant response.

While a Puzzled response can be the appropriate response, for example, when there is no suitable prewritten response or information provision, monitoring the instances when such a response is elicited highlights areas where Anxiety UK can consider extending the response options or the information and services they provide through their website.

### Pattern Matching

In this section, we report some of the modifications made to Ask Anxia designed to attend to features of user queries that can potentially disrupt the pattern-matching mechanism of simple automated chatbot systems. For instance, the Anxiety UK team became aware that the use of certain punctuation affected the ability of the bot to respond correctly to the query and duly updated the program to navigate around such characters.

The simplicity of a pattern-matching procedure is demonstrated when the input (the user query) is not identical to the stimulus the chatbot is programmed to recognize, which can occur with misspellings. In addition to informing us that the term *anxiety* appeared 2461 times in the user queries, the wordlist generated in *#LancsBox* also indicates that the following (likely) misspellings of “anxiety” occurred: *aniety, aniexty, aniexy, anixety, anixity, anixtey, anixty, anxciety, anxeity, anxety, anxiatey, anxiery,* and *anxiey.*

Recognizing common misspellings of relevant terms can help to minimize the number of cases in which the chatbot cannot identify an appropriate response, and while it may be unfeasible to program the service to recognize all possible variants, the wordlist allows us to identify the most common.

The use of negation can result in false negatives, in cases where users produce the relevant stimulus but deny or distance themselves from the concept in their proposition, for example, “not needle phobia.” In such instances, while a chatbot can be programmed to recognize negation (in terms such as *not*, *isn’t*, or *no*), the query does not provide the input to determine what is the impetus of the query, and so recognizing negation would not then help to identify a suitable response. In such cases, the onus may be on the user to deduce how the inappropriate response has been generated (ie, seeing the pattern matching with their original query) and to try reformulating their message. A more proactive response, on the part of the service provider, would be to program the chatbot to recognize negation and to generate a Puzzled-type response that prompts the user to reformulate their query.

Users’ queries might also include additional pattern-matching terms that do not constitute the primary focus of their message but which nevertheless prompt a response This was often the case with longer, more complex query formulations in which multiple competing trigger terms appeared. In most cases, one of the terms would elicit a corresponding response, but this might simply be a greeting to a query that happened to begin with the word *hello*. AbuShawar and Atwell [15] compare a “first-word” approach to a “most significant word” approach with respect to programming chatbots; they explain that the “most significant” word is determined according to low frequency, on the basis that a low-frequency word is what distinguishes an utterance and will favor informational content over high-frequency function words, such as *a*, *to*, *in,* etc. This approach increases the probability that the tool is responding to a “topic” word rather than, say, a grammatical word; however, implementation as part of a simple, pattern-matching chatbot would require additional programming. In the case of Ask Anxia, the Anxiety UK team introduced a prompt in August 2022 that advised users to construct their queries in a simple and direct manner, thereby maximizing the potential for Ask Anxia to recognize a relevant term. Such a response can be generated on the basis of the length of the query (ie, character or line count).

### Managing Expectations

In the previous section, we have seen that optimizing a chatbot service relies, to some extent, on the understanding of the user that, for example, simple direct queries are likely to produce the best results. As such, there is a degree of familiarity, or “literacy,” that can help to ensure that users find the support and information that is of most benefit to them. Working toward this alignment between user goals and service is also a case of managing expectations, first and foremost in relation to what the Ask Anxia service is and can do.

A series of updates applied to Ask Anxia reflected the increasing explicitness with which the Anxiety UK team described the automated nature of the service. Shortly after its initial launch (July 2021), the team supplemented the initial “Hello” response with the following message:

Hello, I am Anxia the Anxiety UK chat bot. I am here to provide you with advice and information. A brief disclaimer: this is not a crisis service, if you feel you are at risk, please contract 111 or 999. Now, how may I help?

Subsequently, Ask Anxia has been relabeled an “eHelper” (August 2021) to avoid potential stigma associated with “chatbots” [3], then later (February 2022), the introductory prompt was rephrased to read the following: “Ask Anxia—Not human but here to help.” Reviewing user queries, it becomes apparent why this clarification that the service is not operated by a human was required.

In Table 2, we say that one of the keywords for user queries was *oh*. Heritage [16] asserts that “where oh is produced as a response to information of some kind, it functions as a ‘change of state’ token; it registers, or at least enacts the registration of, a change in its producer’s state of knowledge or information.” In other words, the use of *oh* can indicate a degree of surprise or unexpectedness on the part of the recipient. When we refer to the queries, we see that often, this interjection reflected a realization on the part of the user that they were interacting with an automated service. The wordlist for the queries demonstrates the number of references to *bot* (87), *robot* (62), and *chatbot* (3), and we can extend our analysis in *#LancsBox* to determine frequencies of fixed phrases that include these terms, namely, *are you a robot* (21), *is this a bot* (16), *is this a robot* (14), *are you a bot* (12), etc.

**Figure 2:**
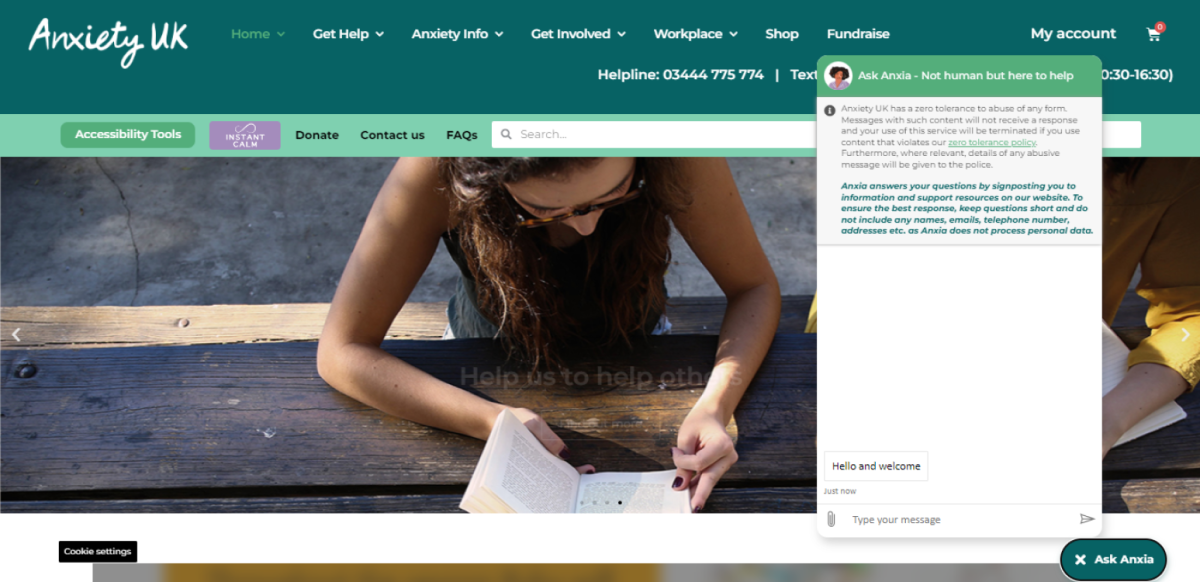
The Ask Anxia chat window as it appears on the Anxiety UK website.

On the one hand, this realization indicates a prior belief that the service was operated by a human and thereby might attest to the verisimilitude of the responses. On the other hand, the fact that this realization has come about indicates that such an illusion has been shattered, that is, because of an inappropriate response or perhaps because of repetition of the kind that is associated with pattern-matched chatbots with a limited number of responses [17]. Thus, with the aim of managing expectations and minimizing the potential for interactional trouble, the Anxiety UK team has worked toward more explicit signaling of the automated nature of the service (Figure 2). With this transparency, users can design their queries appropriately, and Anxiety UK can avoid too many instances where users become disillusioned by the potentially jarring realization that the interaction is not what they had presumed. Furthermore, we have established that some users may be more forthcoming knowing they are interacting with a nonhuman automated service [6].

The Anxiety UK team had initially programmed Ask Anxia with a small number of light-hearted, conversational responses that contributed to a kind of persona, such as “I’m great, thanks for asking!” and “I don’t have an age.” Many of these responses were removed around September 2021 to October 2021, as they were often generated in inappropriate situations and there was a danger that they undermined the serious nature of the user query. Following their survey of motivations for using medical chatbots, Chang et al [3] determined that helping users acquire critical health information should take precedence over whether or not the chatbot appears empathetic or personable. Furthermore, when chatbots appear “humanlike,” this can raise expectations about interactive capabilities, which in turn can negatively impact the interaction when the service’s limitations are exposed [18]. Ultimately, transparency around the service’s purpose and capabilities can help to avoid communicative misalignments and, given the high risk for responses that are designed to appear person like to actually appear flippant, such responses are arguably best avoided when user disengagement could potentially give rise to detrimental health consequences.

Finally, while Anxiety UK encourages users to be candid in their queries, on the basis that being direct will most likely mean that they can get the appropriate support, another series of developments to the Ask Anxia service has been to communicate a zero-tolerance policy for the use of profanities or abuse. A predefined response expressing this position was introduced in December 2021 and has since been subject to minor edits (also shown in Figure 2). Whether the use of profane language is motivated by frustration or is a more facetious “test” of the chatbot’s capabilities (and there is evidence in the queries to indicate both), this does not include terms that are likely to be pattern matched to an informational resource. As such, there is no more preferable response for Ask Anxia to provide, other than to restate the zero-tolerance policy for such language, or alternatively, a response that encourages the user to reformulate their query. A summary of the updates described here is provided in Table 4.

**Table 4:** Summary of updates to the Ask Anxia service.

Date	Action
July 2021	Extended welcome message clarifying that the service is operated by a bot as opposed to a human and to advise users that the service is not a crisis service.
August 2021	Removed the limit cap on messages.
September 2021	Removed a selection of prebuilt answers that were designed to make the bot seem more friendly and human-like but were giving inappropriate responses to sensitive queries. Added a response advising users that the bot did not know how to answer their query (“Sorry I’m not sure how to answer that...”) as an alternative to generating poorly matched responses from the existing content bank.
December 2021	Added a “zero-tolerance policy” response due to a minority of users using profanities. Further clarification of this response was enacted through minor amendments in January 2022 and also February 2022.
July 2022	Added a response discouraging users from including personal data in their queries.
August 2022	Added a response advising users to keep messages brief, on the basis that longer queries gave rise to confusion and poor responses from the chatbot.
October 2022	Updated the content bank to recognize regular typing errors related to existing prompt terms.

### Future Developments

It is worth highlighting some of the anticipated developments that will be implemented to continue to optimize the Ask Anxia service. These developments primarily orient around connecting users to the appropriate mode of service, for instance, providing the connection to contact a (human) operator when this is recognized in the user query. There are also instances in which a more informed response, beyond the level of detail provided in the preprogrammed replies, is required; in such cases, where the user query seems to rely upon more specific contextual or personal circumstances, Ask Anxia can direct users toward the helpline. The Anxiety UK team continues to refine the Puzzled responses to encourage further engagement from the user, for instance, providing the prompt, “Can you phrase this differently?” Finally, the Anxiety UK team is working on developing a mobile app that has the chatbot functionality embedded within it, thereby providing another arm of support and format to use the Ask Anxia service to reach a wider audience and attend to different user preferences.

## Discussion

Organizations implementing pattern-matching chatbots for the purposes of providing information and support will benefit from continuous review of the response options and queries that users submit to their service. Furthermore, an initial set of programmed responses will likely need to be extended, and this will be informed by the nature of the queries that users submit. Our corpus analysis of frequently used terms in user queries to Ask Anxia demonstrated that the initial set of programmed responses was well aligned with the concerns of users but nevertheless helped to highlight areas where additional materials could prove to be useful. The manual quality coding of responses showed that Ask Anxia performs well, offering Good responses at a rate consistently >60%, and this procedure helped to identify areas where responses could be developed to address information gaps or otherwise refined to discern, for example, queries about needles generally and questions about specific vaccinations.

With respect to lessons learned through the implementation and review of the service, first, we have highlighted the informal nature of user queries, which often included ritualized greetings (*Hi* and *hello*). As such, it is useful to have a chatbot response that simply provides a greeting in kind. However, it is important to note that if a user greeting appears at the beginning of a more elaborate query, a response that attends to the topic of the query would be more appropriate.

Second, we have recommended that when an appropriate response cannot be readily identified, there is value in continuing the exchange, that is, encouraging the user to reformulate their query and thereby create additional input from which the chatbot can match an appropriate response. Researchers have highlighted the dangers of “nonprogress” responses that result in user disengagement [13]. Thus, while service providers are unlikely to be able to anticipate the full range of queries their users will submit, they can at least work to facilitate further engagement and use a preprogrammed, albeit uncertain reply to instruct participants on how best to elicit an acceptable response.

Third, we have seen that it is important to manage users’ expectations about what the tool can provide, which includes being explicit that the service is not provided by a human. Relatedly, responses that presented humanlike qualities proved to be of limited value, potentially raising expectations that the tool could offer humanlike judgments. Simple, pattern-matching chatbots such as Ask Anxia are best suited to “frequently asked questions”–type services, rather than more interactional, relationship-building tasks [17]. The benefit of these less-complex systems is that they are easier to program and implement and so can be adopted by service providers with minimal knowledge of the computational systems involved. It is important, nevertheless, to be cognizant of the limitations of such services. For instance, Ask Anxia does not track conversations over multiple turns but rather treats each post as a new query; as such, any pertinent information provided at a previous turn is lost, and users may find themselves having to restate the fundamental purpose of their query. Similarly, the quality of Ask Anxia’s performance is likely to diminish with longer, more complex queries, as it becomes more difficult to discern a singular, relevant prompt. Subsequently, users will be discouraged from providing contextual information (Figure 2) and are unlikely to receive personalized support in this mode. Simple chatbots, therefore, are arguably best used as part of an array of support options, including those which allow for more nuanced exchanges, for example, with a human provider over the telephone.

Laranjo et al [1] assert, based on a systematic review, that applications of chatbots in health care are in the early stages of development and evaluation. Furthermore, the systems used in health care lag behind those used in domains such as travel information and restaurant selection. As their deployment can have consequences for health outcomes, it is appropriate that such systems are continuously tested and evaluated. Language analysis is key to understanding both how users express themselves in queries to chatbots and the design of appropriate responses, and so, we advocate for the continued application of procedures such as those of corpus linguistics to support the extended use and performance of chatbots in health care.

## Conclusions

The launch of the chatbot Ask Anxia was designed to support Anxiety UK in delivering information and support services to people concerned with anxiety disorders. The number of queries submitted to Ask Anxia, particularly out of hours, attests to the value of the service. In this study, we have demonstrated that procedures from corpus linguistics can help to identify patterns in user queries that reflect their needs and expectations of the service as well as direct us to where potential breakdowns in communication occur. For chatbot services to achieve optimal performance, human oversight is required, particularly during the first 6 to 12 months. Thereafter, less staff intervention is likely to be needed.

## References

[1] Laranjo L, Dunn AG, Tong HL, Kocaballi AB, Chen J, Bashir R, Surian D, Gallego B, Magrabi F, Lau AY, Coiera E (2018). Conversational agents in healthcare: a systematic review. J Am Med Inform Assoc.

[2] Nadarzynski T, Miles O, Cowie A, Ridge D (2019). Acceptability of artificial intelligence (AI)-led chatbot services in healthcare: a mixed-methods study. Digit Health.

[3] Chang IC, Shih YS, Kuo KM (2022). Why would you use medical chatbots? Interview and survey. Int J Med Inform.

[4] Amiri P, Karahanna E (2022). Chatbot use cases in the COVID-19 public health response. J Am Med Inform Assoc.

[5] Tudor Car L, Dhinagaran DA, Kyaw BM, Kowatsch T, Joty S, Theng YL, Atun R (2020). Conversational agents in health care: scoping review and conceptual analysis. J Med Internet Res.

[6] Palanica A, Flaschner P, Thommandram A, Li M, Fossat Y (2019). Physicians' perceptions of chatbots in health care: cross-sectional web-based survey. J Med Internet Res.

[7] Vaidyam AN, Wisniewski H, Halamka JD, Kashavan MS, Torous JB (2019). Chatbots and conversational agents in mental health: a review of the psychiatric landscape. Can J Psychiatry.

[8] Baker P, Hardie A, McEnery T (2006). A Glossary of Corpus Linguistics.

[9] American Psychiatric Association (2013). Diagnostic and Statistical Manual of Mental Disorders. 5th edition.

[10] McManus S, Bebbington P, Jenkins R, Brugha T (2016). Mental health and wellbeing in England: adult psychiatric morbidity survey 2014. NHS Digital.

[11] Brezina V, Weill-Tessier P, McEnery A (2021). #LancsBox v.6. Lancaster University.

[12] Love R, Dembry C, Hardie A, Brezina V, McEnery T (2022). The Spoken BNC2014: designing and building a spoken corpus of everyday conversations. Int J Corpus Linguist.

[13] Li CH, Yeh SF, Chang TJ, Tsai MH, Chen K, Chang YJ (2020). A conversation analysis of non-progress and coping strategies with a banking task-oriented chatbot. Proceedings of the 2020 CHI Conference on Human Factors in Computing Systems.

[14] Myers C, Furqan A, Nebolsky J, Caro K, Zhu J (2018). Patterns for how users overcome obstacles in voice user interfaces. Proceedings of the 2018 CHI Conference on Human Factors in Computing Systems.

[15] AbuShawar B, Atwell E (2015). ALICE chatbot: trials and outputs. Comp Y Sist.

[16] Heritage J (1998). Oh-prefaced responses to inquiry. Lang Soc.

[17] Boucher EM, Harake NR, Ward HE, Stoeckl SE, Vargas J, Minkel J, Parks AC, Zilca R (2021). Artificially intelligent chatbots in digital mental health interventions: a review. Expert Rev Med Devices.

[18] Gnewuch U, Morana S, Mädche A (2017). Towards designing cooperative and social conversational agents for customer service. Proceedings of the 2017 International Conference on Information Systems - Transforming Society with Digital Innovation.

